# FeedMaster: A least-cost feed formulation App for minimizing the cost and maximizing milk yield

**DOI:** 10.5455/javar.2022.i605

**Published:** 2022-09-29

**Authors:** Md. Ahsanul Kabir, Nasrin Sultana, Abdullah Al Noman, S. M. Jahangir Hossain, Md. Faizul Hossain Miraz, Gautam Kumar Deb

**Affiliations:** 1 Biotechnology Division, Bangladesh Livestock Research Institute, Savar, Dhaka 1341, Bangladesh; 2 College of Animal Science and Technology, Huazhong Agricultural University, Wuhan 430070, Hubei, China; 3 Director Research, Bangladesh Livestock Research Institute, Savar, Dhaka 1341, Bangladesh; 4 Department of Genetic Engineering Biotechnology, Faculty of Biological Science and Technology, Jashore University of Science and Technology, Jashore, Bangladesh; 5 Training, Planning, and Technology testing Division, Bangladesh Livestock Research Institute, Savar, Dhaka 1341, Bangladesh; † These two authors contributed equally.

**Keywords:** Feeding-assist tool, least-cost ration formulation, mobile app, milk yield, cattle management

## Abstract

**Objective:**

The study aimed to develop and assess an Android app designed for farmers with a low educational status that can formulate a least-cost ration.

**Materials and Methods:**

First, a computer-android-based app named BLRI FeedMaster was developed to guide users in formulating a balanced ration at the least cost. A survey was conducted on 30 livestock officers and 18 farmers with 50 cattle to evaluate its efficacy at the field level. The study outcomes were milk yield, feeding cost, milk composition, time, and cost for management before and after using the BLRI FeedMaster app. Descriptive statistics and paired sample t-tests were used to analyze the data.

**Results:**

After adopting the BLRI FeedMaster app, a significant increase was observed in daily average milk yield (9.39 ± 0.32 l from 8.37 ± 0.36 l), while a considerable decrease was observed in daily average feed quantity (4.88 ± 0.15 kg from 5.60 ± 0.17 kg) and feed cost (BDT 28.00 ± 0.50 from BDT 29.75 ± 0.49). Besides, the number of visits, time, and cost for seeking professional services regarding feed, health care, and other information was significantly minimized. The number of visits decreased to 0.36 ± 013 from 3.07 ± 0.38, and the consumed time was reduced from 270 ± 34.30 to 235.71 ± 59.42 min (*p <* 0.05) after adopting the app.

**Conclusion:**

Hence, this app was very beneficial for farmers with a low economic and educational background and may ultimately help farmers with profitable animal farming and sustainable production in the least developed countries like Bangladesh.

## Introduction

Bangladesh largely depends on livestock for food security, nutritional requirements, and poverty alleviation. About 20% of the population is directly involved with livestock, while another 50% is partly engaged in the livestock sector [1]. According to the Department of Livestock Services, livestock and poultry contributed 1.43% of Bangladesh‘s gross domestic product in 2019–20 [2]. Small households and non-farm households own the majority of livestock and poultry. The share of small households in livestock and poultry constitutes 55.4%, whereas non-farm households possess 20.5% of livestock and poultry [3]. Moreover, livestock, poultry, and related products account for 44% of the animal protein humans consume daily [4].

However, balanced fodder, efficient feeding practices, and intelligent management are necessary for obtaining maximum animal benefits. Other than genetic factors, profitable animal production mainly depends on proper feeding, where feeding costs contribute largely to the overall production cost. Previous studies revealed that feeding cost comprises 70% of the production cost in the dairy and 70%–75% of the production cost in the animal breeding industry [5,6]. Yet, several constraints hamper feeding and fodder management for livestock production in Bangladesh. First, most farmers and livestock owners heavily rely on the nature-derived traditional feeding system that contains little or no concentrates.

On top of that, natural feed resources vary in quantity and quality from season to season and become scarce during the dry season [7]. Second, the complexities of formulating a balanced diet have made it burdensome. Feed quantity and nutrition values differ according to animals and their physiological and morphological features that require empirical knowledge. Hence, there was less application of scientific methods due to a lack of understanding by farmers regarding the physiological and morphological characteristics of animals, stages of production, and animal health-care management. Moreover, most cattle owners have little knowledge about shelter, feeding, nutrition, disease prevention, and control regarding livestock rearing [8]. In addition, they do not have adequate opportunities for proper training [9]. Manually figuring out a balanced ration is a difficult task, but a computer or mobile app can do any calculation in a minute.

Hence, considering these significant factors, the current study has sketched a scientific, smart, handy tool, namely the BLRI FeedMaster, that can satisfy the objectives of improving a cost-effective production with the minimum available feed ingredients, requiring very little knowledge to operate. Several traditional methods, such as trial-and-error, simultaneous equations, and various novel techniques, including linear and nonlinear programming and least-cost formulation, have been proposed and developed for a balanced feed formulation [10]. The main objective of the BLRI FeedMaster was to develop a least-cost ration without difficulty. It can be found as web-based software and as an Android-based downloadable mobile app from the Google Play Store. Mobile phone and internet access have been increasing in Bangladesh. Many farmers use mobile phones to learn and disseminate their business information. A study by Jamie Anderson et al. showed that 82% of smallholder farmers in Bangladesh had used a mobile phone, while 73% owned one. Sixty-seven percent perceived mobile phones as “very important” and having a beneficial role in agriculture-related services [11]. Although numerous balanced ration formulation tools are available in other countries, the farmers of Bangladesh do not use them due to paid subscriptions, lack of expertise, and language barriers. Since most livestock owners and small-scale farmers have little or no knowledge, these skilled and semiskilled-oriented tools have not been adopted in the country. Therefore, considering the facts above, the FeedMaster tool is designed in a bilingual system (Bangla and English), mainly for livestock owners and smallholder farmers with little knowledge and limited expertise. The BLRI FeedMaster tool has different kinds of information, such as how to make rations based on the weight of the animal, how to grow fodder all year, how to vaccinate, and how to set up an alarm system.

## Materials and methods

### Theoretical basis of the BLRI FeedMaster application

The BLRI Feedmaster app is named after the Bangladesh Livestock Research Institute, the organization that developed it. The application is based on the least-cost formulation method and can formulate rations for cattle instantly. Ration formulation was based on the National Research Council (NRC) guidelines [12,13]. All the animals were categorized into two groups. A ration containing higher crude protein (CP) with no supplement was allocated for one group consisting of growing animals and bulls (Ration A), while a ration containing lower CP with supplements of feed was allocated for another group consisting of dry, pregnant, and lactating animals (Ration B). In terms of growing animals, the ration was designed based on cattle fattening. Moreover, we have developed different rations with the same feed ingredients. We want to make a ration with the feed resources that are available in Bangladesh so that farmers in all parts of the country can benefit. Furthermore, 40 dairy cattle were categorized into five groups according to milk-producing ability, and supplemented concentrate was calculated based on their milk production by using the following formulas [14]:

> 5 M.P (L) = 0.5 + 0.7*X* M.P (L)

> 8 M.P (L) = 0.7 + 0.4*X* M.P (L)

> 12 M.P (L) = 2.0 + 0.4*X* M.P (L)

> 15 M.P (L) = 3.0 + 0.3*X* M.P (L)

> 20 M.P (L) = 3.0 + 0.3*X* M.P (L)

Lactating animals were provided supplementary and basal feed, while pregnant animals were provided supplementary feed after six months of pregnancy. The average dry matter (DM) consumption was calculated as 2.5 % of body weight for all groups of cattle. Concentrate and two-thirds of DM fulfil one-third of DM is fulfilled by roughage among total DM requirements.

### Concentrate

An animal’s body weight is a prerequisite for computing the ration. With this tool, farmers can impute animal body weight directly or use the measuring tape (measuring heart girth and body length in inches). Animal weight was calculated using Schaeffer’s formula [15].

*W* = (*L*) × (HG)^2^/660.

Here *W* = body weight in kg; *L* = Length of the animal in inches (from the point of shoulder to pin bone); HG = Heart girth of the animal in inches.

Maize crush, wheat bran, rice polish, khesari bran, soybean meal, salt, dicalcium phosphate, and limestone were used as concentrates to fulfill the concentrate requirement in the ration. Table 1 lists the ingredients used in the ration formulation.

### Roughage and cultivation

A farmer can fulfill roughage requirements using various seasonal (Maize, Jamboo, Oat, and Dhaincha) and perennial (German and Napier) grasses. The BLRI FeedMaster can compute the amount of roughage required based on the DM content of the fodder. Additionally, this tool can calculate and provide information on the requirements of land, seed/cutting, urea, TSP, MOP, and their cost. These requirements were computed considering the livestock and farm practices of the country. The annual fodder supply was planned based on the seasons and fodder availability in a particular season. The total cultivable land was divided into four groups: three were categorized as highland and one as lowland. Two highlands and one lowland were used for perennial crop cultivation, while a third highland was used for seasonal crops. The land requirement for each plot was calculated in acres using the following formula:

Land requirement (acres) = {Month wise DM requirement of cattle (Weight of the animal × 1.6666 × 31)/DM content of fodder}/ The average production of fodder/hector per cutting × hector conventional factor (2.47).

This formula was developed based on the farm practice of BLRI. Besides, the annual fodder production plan was designed based on the arability of fodder in a season, land type, and production capacity of fodder.

**Table 1.** Amount of feed ingredient use in the ration.

Ingredients	Ration A	Ration B
Maize crush (%)	25	30
Rice polish (%)	20	20
Khesary bran (%)	15	20
Wheat bran (%)	20	15
Soybean meal (%)	17	12
Salt (%)	1	1
Di-calcium phosphate (%)	1.5	1.5
Lime stone (%)	0.5	0.5
CP (%)	19.6	17.22
Energy (MJME/kg)	11.11	11.67

### Software development and features

**Figure 1.**
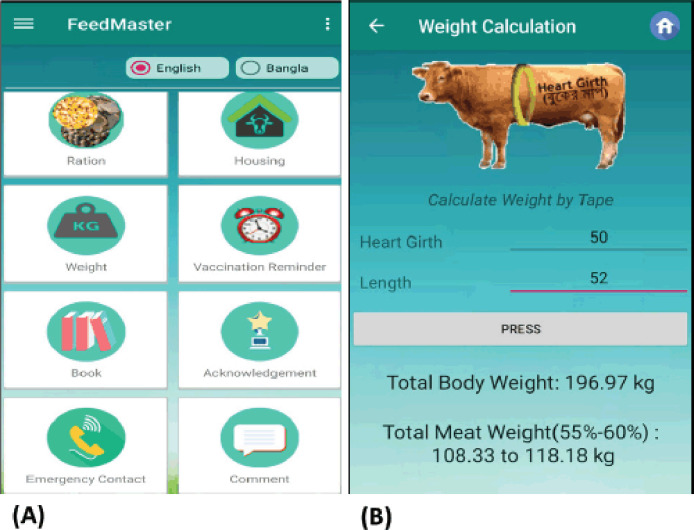
(A) Homepage and (B) weight calculation feature of BLRI FeedMaster.

The BLRI FeedMaster software was built with Java, a high-level programming language. It is one of the most popular software and application development languages due to its accessibility to virtually all devices, including computers and smartphones. A database was created with MS Access for data management, which included information on feed resources and their type, price, and dry matter content. A Visual Basic program was written to calculate a least-cost ration with balanced nutrition according to the category of cattle and locally available feed. We used a bilingual graphical user interface that can compute and provide information, including body weight, estimated meat content, requirements of concentrate and roughage, and annual fodder planning according to the animal category. Besides, users will get information about the estimated feed cost by inputting the price. The homepage of the BLRI FeedMaster illustrates various app features (Fig. 1A). The weight of any animal can be calculated from the “Weight” feature (Fig. 1B).

### Steps for ration formulation

**Figure 2.**
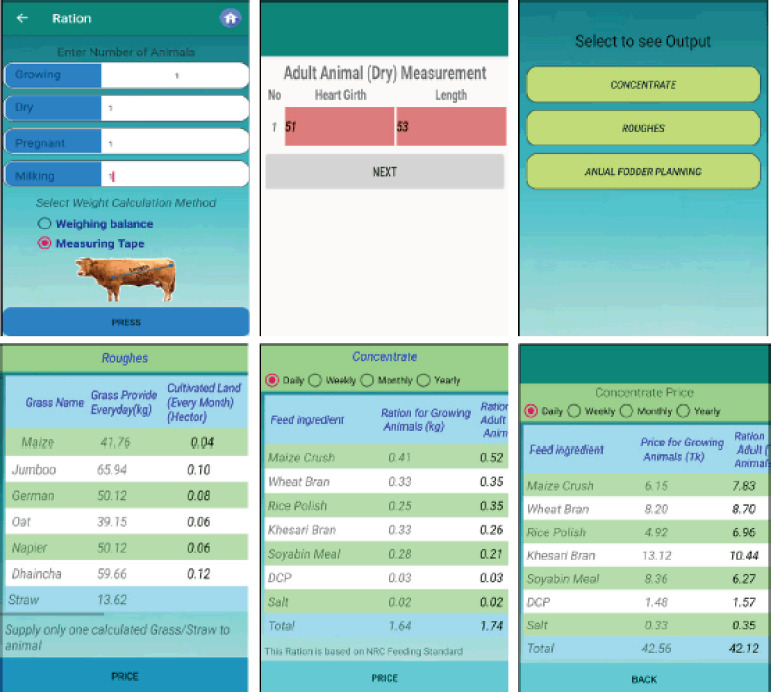
Ration formulation according to cattle’s weight.

Users can formulate a balanced feed from the “ration” feature of the app. After computing the weight of every animal, users need to proceed by clicking on the “Ration” icon that will provide detailed feed requirements along with the quantity of grass, roughage, dry matter content, and cost (Fig. 2).

**Figure 3.**
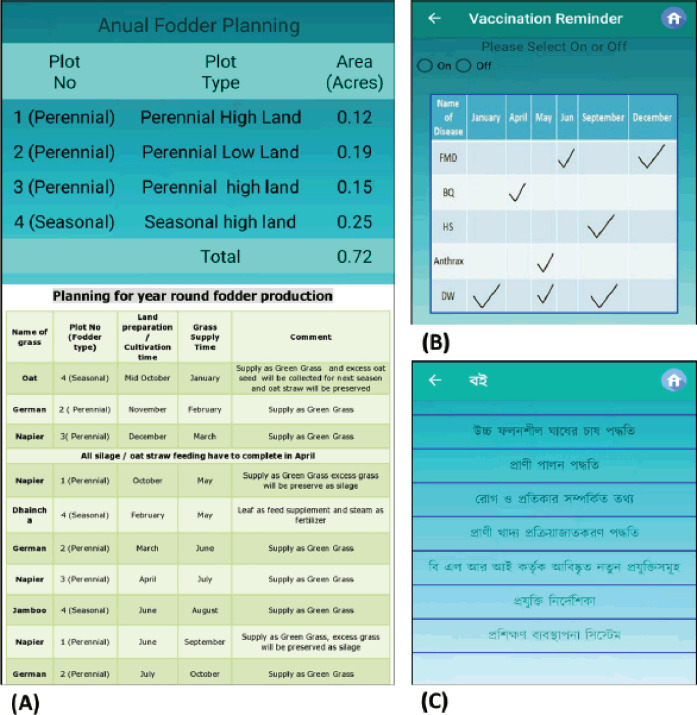
(A) Annual fodder planning, (B) vaccination reminder, and (C) reference books.

1. Selecting the cattle category- The number of cattle followed by bodyweight of cattle is entered according to the cattle category (growing, pregnant, etc.). Bodyweight can be inserted in two ways: Weighing balance and measuring tape (Schaeffer’s formula).
2. Select the output from thecategories of concentrates and roughage to see the least cost ration. To see the estimated cost of a feed, enter the price.
3. Select annual fodder planning to see the estimated land segregation along with year-round fodder cultivation (Fig. 3A).

Other essential features include a vaccination reminder, reference books, and emergency contact. Only balanced nutrition cannot ensure sustainable production. The livestock may remain susceptible to diseases if not vaccinated, and it is an established phrase that “prevention is better than cure.” Thus, a vaccination alarm feature has been added to remind the farmers about vaccination and helminticides of their cattle against viruses and helminths, respectively (Fig. 3B). By clicking the ‘On’ option, the user will be reminded about vaccination on due time via automated SMS free of charge. Information on cattle shelters, methods of high-yield grass production, cattle rearing, diseases and treatments, and feed and fodder processing have been provided in the “Book feature” (Fig. 3C). To obtain maximum benefits with the least cost, one should know about the cultivation of high-yield grass, how to design a shelter, and how to produce feeds for the cattle. Also, livestock owners should be aware of common diseases in a particular species or region. All this information can be found in this feature.

### Assessment of the efficacy of the BLRI FeedMaster app

After developing the FeedMaster app, its competency was evaluated by livestock experts. Three days of training were conducted on using the tool for 30 District Livestock Officers (DLO) working as livestock experts at field level. Their opinions were evaluated through a pretested questionnaire, and necessary modifications were made based on their recommendations. Then, the impact and efficacy of the app on livestock productivity were assessed by conducting a baseline survey. Initially, 50 livestock owners from Godagari Upazila of the Rajshahi district were selected for training on using BLRI FeedMaster, and 21 of them stated positively regarding implementing this application on their farms. Of 21 farmers, 18 participants were selected for data collection, and the cattle population was 40. A regional station of the BLRI is situated at Rajshahi. With the help of a DLO, potential subjects were chosen randomly. They were trained by experts and underwent a six-month follow-up study. A MASTER ECO milk analyzer was used to measure the main milk composition. A questionnaire was designed to assess the milk yield, feed supply, feeding costs, monitoring costs, and the number of visits before and after using the BLRI FeedMaster app.

**Figure 4.**
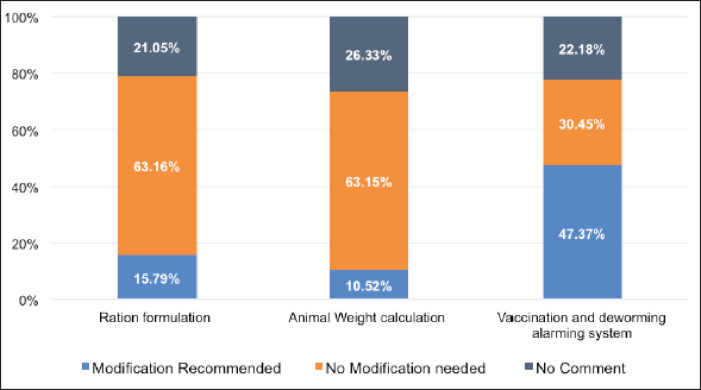
Recommendations of livestock experts on the BLRI FeedMaster.

Descriptive statistics were performed to present frequency, and a paired sample t-test was performed to determine the effect on time cost and the number of visits for collecting necessary information. The *z*-test was conducted to assess milk yield, the quantity of feed supply, feed cost, and milk composition. The data were managed and analyzed by Microsoft Excel 2010 and SPSS (IBM SPSS version 20.0) software.

## Results

### Assessment of the BLRI FeedMaster app by the livestock experts

All livestock experts (100%) recommended the BLRI FeedMaster tool for farmers as a user-friendly technology that might increase productivity with a reduced cost at the field level. Figure 4 shows that only 16% of experts recommended modifications to the ration formulation feature by adding alternative feed ingredient options with robust nutritional composition. Eleven percent of experts recommended adding a meat calculation feature. But experts suggested adding an alarm/reminder system to the vaccination and deworming schedule. All the features are available in the latest version of the app.

### Impacts of the BLRI FeedMaster app on milk yield, feed supply, and feed cost

Our study showed that daily average milk production increased, but the quantity of feed supply and feed cost decreased after using the BLRI FeedMaster ration formulation tool (Table 2). It was observed that milk yield significantly increased (*p <* 0.05) from 8.37 ± 0.36 l/animal/day to 9.39 ± 0.32 l/animal/day after using the tool. However, the quantity of feed supply was markedly reduced (*p <* 0.05) from 5.60 ± 0.17 to 4.88 ± 0.15 kg/day/animal after using the tool, albeit all the ingredients and nutrition levels were the same as before. Moreover, the daily feed cost for formulating a balanced ration per animal significantly decreased (*p <* 0.005) to BDT 28.00 ± 0.50 from BDT 29.75 ± 0.49 after adopting the tool. So, these results proved that using the app increased milk yield while decreasing the amount of feed needed and the cost of feed.

### Impacts of BLRI FeedMaster app on milk composition

The effects of the BLRI FeedMaster app on milk composition were also examined (Table 3). Milk analysis revealed that the fat percentage increased from 4.12 ± 0.13% to 4.91 ± 0.14% but was not statistically significant. (*p* > 0.05). Moreover, there was no statistical difference in other milk compositions such as solid-non-fat (SNF), protein, and lactose before and after adopting the app.

### Impacts of BLRI FeedMaster app on information-seeking behaviour

This study found that after adopting the BLRI FeedMaster, the number of visits, time, and cost for seeking professional service regarding ration, feed, and health-care practices significantly minimized (Table 4). The visits decreased to 0.36 ± 013 from 3.07 ± 0.38 after using the app. The time spent and cost were subsequently reduced. The time that was spent seeking advice was reduced from 270 ± 34.30 to 235.71 ± 59.42 min (*p <* 0.05), and the travel cost was reduced to BDT 3.21 ± 1.51 from BDT 5.29 ± 1.50 after adopting the app.

**Table 2.** Impacts of BLRI FeedMaster tool on milk yield, the quantity of feed supply and feed cost.

Variables	Before adopting the BLRI FeedMaster app (mean ± SE)	After adopting the BLRI FeedMaster app (mean ± SE)	Sig. (2-tailed)
Milk yield (liter/animal/day)	8.37 ± 0.36	9.39 ± 0.32	*
Quantity of feed supply (kg/day/animal)	5.60 ± 0.17	4.88 ± 0.15	*
Feed cost (BDT/day/animal)	29.75 ± 0.49	28.00 ± 0.50	*

* Significant at 5% level of significance.

**Table 3.** Impacts of BLRI FeedMaster tool on milk composition.

Parameters	Before adopting the BLRI FeedMaster app (mean ± SE)	After adopting the BLRI FeedMaster app (mean ± SE)	Sig. (2-tailed)
Fat (%)	4.12 ± 0.13	4.91 ± 0.14	NS
SNF (%)	3.65 ± 0.07	3.67 ± 0.06	NS
Protein (%)	10.26 ± 0.11	10.30 ± 0.12	NS
Lactose (%)	5.39 ± 0.20	5.41 ± 0.21	NS

NS = Non-significant at 5% level of significant.

**Table 4.** Impacts of BLRI FeedMaster tool on time, cost, and number of visits for monitoring.

Parameters	Before adopting the BLRI FeedMaster app (mean ± SE)	After adopting the BLRI FeedMaster app (mean ± SE)	Sig. (2-tailed)
Time (min	270 ± 34.30	235.71 ± 59.42	*
Cost (taka)	5.29 ± 1.50	3.21 ± 1.51	*
Visit (time)	3.07 ± 0.38	0.36 ± 0.13	*

* Significant at 5% level of significance.

## Discussion

To the best of authors’ knowledge, this is the first study in Bangladesh that showed the need and efficacy of an app designed using locally available feed resources for formulating rations according to farmers’ choices. This study found that the BLRI FeedMaster app greatly improved milk production and quality while reducing the feed supply and cost of feed and labor involved in livestock rearing.

Our study demonstrated that after using the FeedMaster app, the daily average milk yield increased by over 1 l/animal/day). The increase in output may be attributed to the elevated rumen function, where enhanced microbial fermentation occurred due to a balanced ration. The FeedMaster-formulated allocation contains all the essential nutrients, and previous studies revealed that optimum nutrition and minerals significantly contributed to milk production [16,17]. However, milk content such as fat, protein, SNF, and lactose did not increase significantly. The possible reasons behind this discrepancy may be negative energy balance and environmental or management factors [18]. Moreover, milk compositions cannot be modified without precise conditions. Although the ration included in the app contains DM and dietary CP, according to NRC, CP was not significantly associated with an increase in milk protein percent but moderately associated with protein yield [12].

Using the BLRI FeedMaster ration formulation tool reduced the amount and cost of feed required for cattle while maintaining all the nutrition. The amount of feed decreased to about 1 kg/animal/day; consequently, the price was also minimized. As discussed earlier, identifying and formulating a balanced ration is a complex process that needs knowledge of nutrition values according to animals and their physiological and morphological features. Recent socio-economic studies found that most farmers were illiterate or had completed primary education, ranging from 50% to 75% [19,20]. The BLRI FeedMaster was designed considering local farmers‘ education status and knowledge and therefore assisted farmers in formulating a ration without expert knowledge. Moreover, it significantly minimized the cost of feed. The shortage of different feed ingredients is a serious obstacle that results in higher feed costs. For instance, only 53.5% of the total fibrous biomass produced was available to the cattle, and the country had a deficit of 80% of dry matter and 71.2% of digestible crude protein [21]. Furthermore, 89% of the available roughage is fibrous and contains a minimal amount of digestible crude protein, while a 79.8% deficit exists in required concentrate production. Therefore, farmers in the country have to import the bulk of their required feed. Talukder et al. [19] showed that feed cost constituted about 93% of the total production cost. Since most people involved with livestock rearing in Bangladesh are poor, feed costs impose a considerable strain on them to achieve profitable production.

This study found that after adopting the BLRI FeedMaster, farmers’ dependency on veterinary professionals considerably decreased due to time and cost minimization. The unavailability of timely information and the unwillingness of proper implementation by farmers are significant constraints in the livestock production of Bangladesh and hamper profitability [22,23]. As already discussed, the majority of farmers in the country are illiterate or have very poor knowledge of agriculture inputs such as feeds, nutrition, health-care practices, housing, and agricultural technologies. The information-seeking behavior of farmers mostly depends on the difficulty of the problem and the availability of reliable information [22]. They rely on professional veterinarians or government livestock organizations, which are scarce in many districts and cost extra time and money [24,25]. We found that before using the BLRI FeedMaster app, farmers had to visit three times to seek professional assistance, while after using the app, it was reduced to less than once. The cost and consumed time for travel and service were also decreased subsequently. Using the app saves about 30 min. The app has a step-by-step procedure for developing a balanced ration with the locally available feeds. Moreover, it includes information on estimated feed costs, diseases and vaccinations, land requirements, housing, and common diseases in the Bangla language, which helps a farmer to stay informed. Access to and utilization of scientific information might result in increased and sustainable livestock production. Using scientific knowledge can alleviate farmers‘ lack of knowledge, and this app might be helpful. Above all, these contribute to reducing farmers‘ time, cost, and labor. Therefore, it can be concluded that the BLRI FeedMaster mobile app can benefit poor farmers and livestock owners by helping them formulate a least-cost ration without difficulty and by educating them on rudimentary information about cattle management. It also demonstrates the willingness or satisfaction of farmers concerning the BLRI FeedMaster app. The app was uploaded to the Google Play Store and was downloaded more than 20,000 times from 15 different countries in the world, mainly from Bangladesh (13998), Saudi Arabia (637), and India (571). It received a 4.8 rating out of 5 from users.

Bangladesh is one of the major agriculture-producing countries, and livestock or livestock-related products contribute primarily to the income of the smallholder people as well as the country’s economy. Many rural men and women are involved in cattle rearing, and many consider this the only source of income. Yet, most of them do not obtain maximum profit owing to feeding the cattle with less nutritious rations, high feed costs, lack of knowledge of appropriate feeding and management practices, and improper scientific knowledge of cattle production. But the BLRI FeedMaster is built to provide user-friendly features that will help smallholder farmers formulate a least-cost balanced ration with limited available feed resources that require little knowledge and expertise. Nowadays, fodder cultivation is a great concern in Bangladesh because of the lack of pastureland. The scarcity of quality fodder and inefficient feeding practices are thus considered one of the most significant constraints in obtaining the maximum return from livestock rearing and their expected development. Precision feeding with balanced nutrition will increase the economic, health, and environmental benefits while providing sustainability to livestock and agriculture.

## Conclusion

Profitable livestock production depends on management practices, feeding, scientific approaches, and technologies. The BLRI FeedMaster Android app is a user-friendly mobile application that may help both small households and non-farm households. Using the app at the field level increased milk yield while markedly reducing the quantity of feed supplies and feed costs. Moreover, adopting the tool reduced time, prices, and the number of visits for monitoring. Therefore, this tool might be helpful for livestock owners or farmers from low-income or educational backgrounds by providing a least-cost ration formulation approach that will ultimately result in sustainable production.

## List of abbreviations

BLRI: Bangladesh Livestock Research Institute; CP: Crude Protein; DM: Dry Matter; MOP: Muriate of potash; MJME/KG: Mega Joule Metabolized Energy/kg; NRC: National Research Council; SNF: Solid-non-fat; TSP: Triple superphosphate.
